# Supplementary material for: Robust whole slide image analysis for cervical cancer screening using deep learning
Source: Nat Commun. 2021 Sep 24;12:5639. doi: 10.1038/s41467-021-25296-x (PMC8463673; doi:10.1038/s41467-021-25296-x)
Supplement: Supplementary file 1 — Supplementary Information [file 41467_2021_25296_MOESM1_ESM.pdf]

Robust Whole Slide Image Analysis for Cervical Cancer Screening Using Deep Learning

Supplementary

The architecture of the LR model and generated heatmaps. For the LR model, we modify the fully connected layer of original ResNet50 and add a semantic segmentation branch for generating a rough location mask. As shown in Supplementary Figure 1, the semantic segmentation branch consists of three atrous convolutional blocks and two plain convolutional layers. The atrous convolutional block is composed of a 1 × 1 convolutional layer, a 3 × 3 atrous convolutional layer with a dilation rate of 2 and a 1 × 1 convolutional layer. Thus, the LR model can classify image tiles and locate suspicious lesion areas at the same time. The LR model accepts an image tile of 512 × 512 pixels (0.486 μm/pixel) as input and outputs a lesion probability and a location heatmap. Supplementary Figure 2 shows the merged images of the cervical cell images and corresponding location heatmaps. The suspicious lesion cells can be accurately located, which provides a basis for cropping the HR model's input images of 256 × 256 (0.243 μm/pixel).

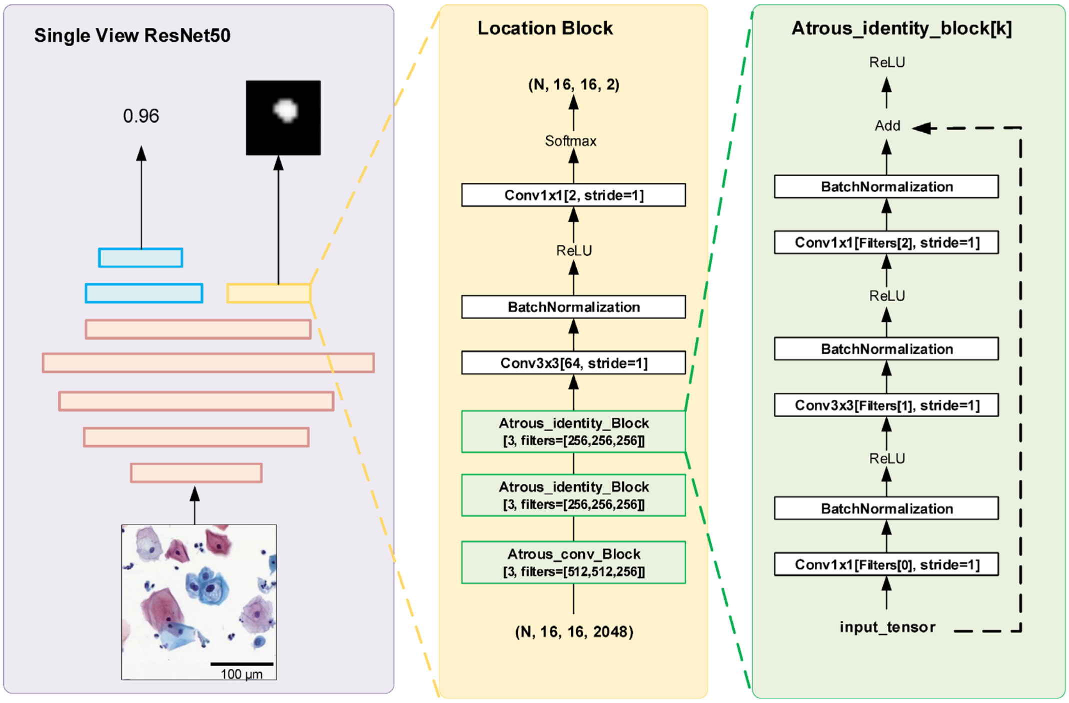

Supplementary Figure 1 | The architecture of the LR model. The LR model consists of a ResNet50 encoder, a classification head and a semantic segmentation head. The semantic segmentation branch is constructed with residual blocks of dilated convolutions. The LR model accepts an image tile of 512 × 512 pixels (0.486 μm/pixel) as input and outputs a lesion probability and a location heatmap.

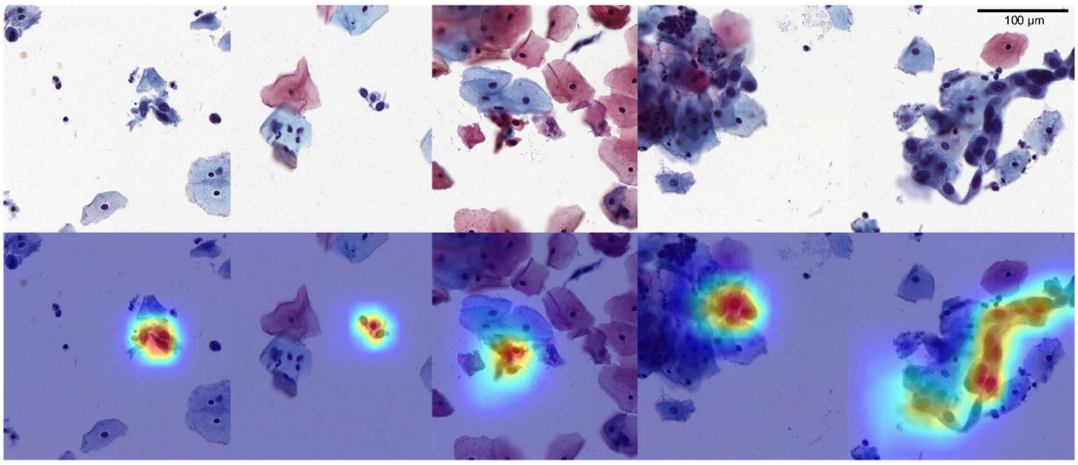

Supplementary Figure 2 | Visualization of heatmaps generated by the LR model. The above are cervical cell image tiles of 512 × 512 pixels (0.486 µm/pixel). The below are the merged images of the cervical cell images and corresponding location heatmaps. Notably, the redder the heatmap, the higher the lesion probability.

The performance of our system among HPV-positive women. To test the performance of our system in the case of HPV testing first or in combination with cytology, we further analyzed the cervical cell slides from 395 HPV-positive women (cytology positive 169, negative 226). These glass slides were gathered from Maternal and Child Hospital of Hubei Province and were scanned into WSIs by the second version instrument from Wuhan National Laboratory for Optoelectronics-Huazhong University of Science and Technology with 0.247 μm/pixel under 20× magnification. The quantitative results of the automated prescreening among these HPV-positive women are shown in Supplementary Figure 3. Supplementary Figure 3a shows 81.9% Specificity and 79.3% Sensitivity with 0.890 AUC (the area under ROC) in case of slides from the 395 HPV-positive patient-wise WSIs. Supplementary Figure 3b shows the frequency histogram of positive and negative slide scores. The main reason for the performance deterioration is that many of these HPV-positive and cytology-negative samples are accompanied by bacterial infections, which may increase the classification difficulty.

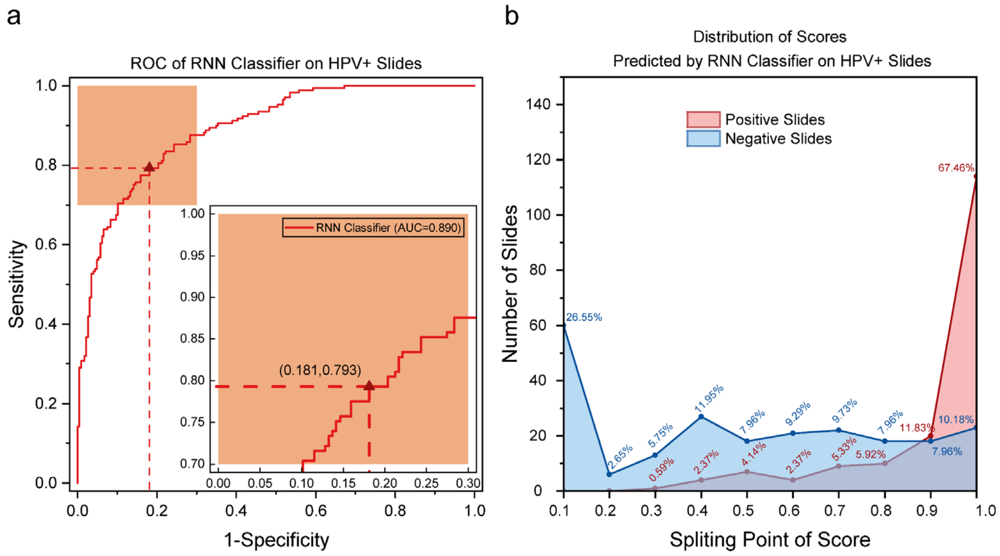

Supplementary Figure 3 | The quantitative results of our WSI analysis system among HPV-positive women. **a**, The ROC of the RNN model for classifying the 395 HPV-positive patient-wise WSIs (cytology positive 169, negative 226). **b**, The frequency histogram of slide scores from 0 to 1 with an interval of 0.1 of the 395 slides (n = 395). The blue and pink areas refer to negative and positive slides respectively.
