# Supplementary material for: Robust whole slide image analysis for cervical cancer screening using deep learning
Source: Nat Commun. 2021 Sep 24;12:5639. doi: 10.1038/s41467-021-25296-x (PMC8463673; doi:10.1038/s41467-021-25296-x)
Supplement: Supplementary file 3 — Reporting Summary [file 41467_2021_25296_MOESM3_ESM.pdf]

nature research

Corresponding author(s): Li Chen, Junbo Hu, Xiuli Liu

Last updated by author(s): Jul 28, 2021

Reporting Summary

Nature Research wishes to improve the reproducibility of the work that we publish. This form provides structure for consistency and transparency in reporting. For further information on Nature Research policies, see our Editorial Policies and the Editorial Policy Checklist.

Statistics

For all statistical analyses, confirm that the following items are present in the figure legend, table legend, main text, or Methods section.

n/a	Confirmed	
☐	☒	The exact sample size (n) for each experimental group/condition, given as a discrete number and unit of measurement
☐	☒	A statement on whether measurements were taken from distinct samples or whether the same sample was measured repeatedly
☒	☐	The statistical test(s) used AND whether they are one- or two-sided<br>*Only common tests should be described solely by name; describe more complex techniques in the Methods section.*
☒	☐	A description of all covariates tested
☒	☐	A description of any assumptions or corrections, such as tests of normality and adjustment for multiple comparisons
☐	☒	A full description of the statistical parameters including central tendency (e.g. means) or other basic estimates (e.g. regression coefficient) AND variation (e.g. standard deviation) or associated estimates of uncertainty (e.g. confidence intervals)
☒	☐	For null hypothesis testing, the test statistic (e.g. F, t, r) with confidence intervals, effect sizes, degrees of freedom and P value noted<br>*Give P values as exact values whenever suitable.*
☒	☐	For Bayesian analysis, information on the choice of priors and Markov chain Monte Carlo settings
☒	☐	For hierarchical and complex designs, identification of the appropriate level for tests and full reporting of outcomes
☒	☐	Estimates of effect sizes (e.g. Cohen's d, Pearson's r), indicating how they were calculated

Our web collection on statistics for biologists contains articles on many of the points above.

Software and code

Policy information about availability of computer code

Data collection	These glass slides are scanned into WSIs by the instruments from 3DHisTech Ltd. with 0.243 µm/pixel under 20× magnification, Shenzhen Shengqiang Technology Ltd. with 0.180 µm/pixel under 40× magnification, Wuhan National Laboratory for Optoelectronics-Huazhong University of Science and Technology with 0.293 µm/pixel under 20× magnification, Huaiguang Intelligent Technology Ltd. with 0.238 µm/pixel under 20× magnification and Konfoong Biotech Information Ltd. with 0.238 µm/pixel under 40× magnification. The cervical WSIs are annotated by six cytopathologists using Qupath software (v0.2.0).
Data analysis	The source codes of the paper is available at https://github.com/ShenghuaCheng/Aided-Diagnosis-System-for-Cervical-Cancer-Screening. The C++ software with a user manual and test data is available at Baidu Cloud (https://pan.baidu.com/s/1UmQzASwvlpKLO7hbwaDc_A, extracting code is cyto) or at Google Drive (https://drive.google.com/drive/folders/19rE9atLrylaBR8shqAlc4Sf8tn62o7ky?usp=sharing, no extracting code).

For manuscripts utilizing custom algorithms or software that are central to the research but not yet described in published literature, software must be made available to editors and reviewers. We strongly encourage code deposition in a community repository (e.g. GitHub). See the Nature Research guidelines for submitting code & software for further information.

Data

Policy information about availability of data

All manuscripts must include a data availability statement. This statement should provide the following information, where applicable:

- Accession codes, unique identifiers, or web links for publicly available datasets
- A list of figures that have associated raw data
- A description of any restrictions on data availability

The LR model and HR model of our progressive lesion cell recognition method were initialized with the pre-training weights of ImageNet (https://www.image-

net.org). The source data underlying Figures 3-7 is provided in the supplementary as SourceData.zip. We further provided the source codes, the C++ software and some test WSIs to facilitate the reproducibility. The original WSI and annotation data are private and are not publicly available since the protection of patients' privacy in cooperative hospitals. All data supporting the findings of this study are available on requests for non-commercial and academic purposes from the primary corresponding author (xlliu@mail.hust.edu.cn) within 10 working days. We do not require to sign a data use agreement.

Field-specific reporting

Please select the one below that is the best fit for your research. If you are not sure, read the appropriate sections before making your selection.

- [x] Life sciences
- [ ] Behavioural & social sciences
- [ ] Ecological, evolutionary & environmental sciences

For a reference copy of the document with all sections, see nature.com/documents/nr-reporting-summary-flat.pdf

Life sciences study design

All studies must disclose on these points even when the disclosure is negative.

Sample size	No sample-size calculation was performed. We collected 12 groups of datasets from 5 hospitals and 5 kinds of imaging instruments. These 12 datasets listed in Figure 2a include 1,467 (41.4%) positive WSIs and 2,073 (58.6%) negative WSIs with 79,911 annotated lesion cells. The data size and data diversity are sufficient for this research.
Data exclusions	We excluded few unsatisfied WSIs in advance that are completely blurred caused by imaging defocus. The exclusion criteria was pre-established.
Replication	The training groups A-D were randomly divided into training set, validation set and test set with a slide-wise ratio of 8: 1: 1 (Figure 2d). The LR model, HR model and RNN model were trained and tested on these data. The models were evaluated on multi-center independent test sets of 1,170 patient-wise WSIs, and compared with three three independent cytopathologists in multiple evaluation metrics, including tile classification accuracy, WSI classification sensitivity and specificity, AUC-ROC. All the experiments can be repeated. We provided the source data of figures in the manuscript and the source codes, the C++ software and some test WSIs to maximize the reproducibility of our method and results (https://github.com/ShenghuaCheng/Aided-Diagnosis-System-for-Cervical-Cancer-Screening and https://pan.baidu.com/s/1UmQzASwvlpKLO7hbwaDc_A, extracting code is cyto).
Randomization	WSI samples were randomized fairly into training, validation and testing datasets.
Blinding	The slide acquisition is performed in accordance with the guidelines of the Medical Ethics Committee of Tongji Medical College at Huazhong University of Science and Technology. No investigator blinding is necessary since our experiments are based on digital slide images and software analysis.

Reporting for specific materials, systems and methods

We require information from authors about some types of materials, experimental systems and methods used in many studies. Here, indicate whether each material, system or method listed is relevant to your study. If you are not sure if a list item applies to your research, read the appropriate section before selecting a response.

Materials & experimental systems

n/a	Involved in the study	
☒	☐	Antibodies
☒	☐	Eukaryotic cell lines
☒	☐	Palaeontology and archaeology
☒	☐	Animals and other organisms
☒	☐	Human research participants
☒	☐	Clinical data
☒	☐	Dual use research of concern

Methods

n/a	Involved in the study	
☒	☐	ChIP-seq
☒	☐	Flow cytometry
☒	☐	MRI-based neuroimaging
